# Dissecting the function of the adult β-globin downstream promoter region using an artificial zinc finger DNA-binding domain

**DOI:** 10.1093/nar/gku107

**Published:** 2014-01-31

**Authors:** Joeva J. Barrow, Ying Li, Mir Hossain, Suming Huang, Jörg Bungert

**Affiliations:** Department of Biochemistry and Molecular Biology, Center for Epigenetics, Genetics Institute, Shands Cancer Center, Powell-Gene Therapy Center, University of Florida, Gainesville, 32610, FL, USA

## Abstract

Developmental stage-specific expression of the β-type globin genes is regulated by many *cis*- and *trans*-acting components. The adult β-globin gene contains an E-box located 60 bp downstream of the transcription start site that has been shown to bind transcription factor upstream stimulatory factor (USF) and to contribute to efficient *in vitro* transcription. We expressed an artificial zinc finger DNA-binding domain (ZF-DBD) targeting this site (+60 ZF-DBD) in murine erythroleukemia cells. Expression of the +60 ZF-DBD reduced the recruitment and elongation of RNA polymerase II (Pol II) at the adult β-globin gene and at the same time increased the binding of Pol II at locus control region (LCR) element HS2, suggesting that Pol II is transferred from the LCR to the globin gene promoters. Expression of the +60 ZF-DBD also reduced the frequency of interactions between the LCR and the adult β-globin promoter. ChIP-exonuclease-sequencing revealed that the +60ZF-DBD was targeted to the adult β-globin downstream promoter and that the binding of the ZF-DBD caused alterations in the association of USF2 containing protein complexes. The data demonstrate that targeting a ZF-DBD to the adult β-globin downstream promoter region interferes with the LCR-mediated recruitment and activity of Pol II.

## INTRODUCTION

Transcription of the adult β-type globin gene is tightly regulated by distal and proximal *cis*-regulatory DNA elements (1,2). Although proximal promoter elements govern developmental-stage and tissue-specific expression of the β-type globin genes, a locus control region (LCR), located far upstream of the genes, mediates high-level expression in differentiating erythroid cells (3–5). The LCR is composed of multiple DNase I hypersensitive sites (HS) that each interact with a combination of ubiquitously expressed or more cell type–restricted transcription factors (6). Among the proteins that bind to the LCR are the hematopoietic transcription factors GATA-1 and -2, NF-E2 and related proteins, KLF1 and related proteins, Tal1 and the ubiquitously expressed proteins USF and Sp1 (6,7). The mechanisms by which the LCR mediates high-level transcription of the β-globin genes are not completely understood. It is known that during differentiation of erythroid cells the genes come in proximity to the LCR (8,9). Furthermore, active chromatin marks and transcription complexes first associate with the LCR during differentiation of erythroid cells (10–13). This suggests that the LCR serves as the primary site of transcription complex recruitment and possibly assembly, and that the assembled transcription complexes are transferred to the globin genes (14). This model is supported by *in vitro* transfer experiments, however; *in vivo* evidence is lacking (15).

The stage-specific expression of the adult β-globin genes is mediated by transcription factors that bind to proximal regulatory elements (16). KLF1 binds to a CACCC site in the upstream promoter and activates β-globin gene expression by recruiting chromatin modifying activities and components of the basal transcription apparatus (17–19). GATA factors have also been shown to recruit chromatin modifying coregulators and activities that establish proximity between the LCR and the adult β-globin gene promoter (20,21). In contrast to these well-established transcription factors, the role of the E-box binding proteins Tal1 and USF are less well understood. Tal1/SCL is a hematopoietic-specific bHLH protein that interacts with ubiquitous bHLH proteins such as E2A and HEB and binds E-box elements (22). In erythroid cells, Tal1 is part of a large transcription factor complex that includes GATA-1, its cofactor Fog1 and the cofactors Ldb1 and LMO2 (23). There is evidence showing that Ldb1 is important for establishing proximity between the LCR and the adult β-globin gene promoter (24,25). Tal1 can be cross-linked to the adult β-globin promoter in erythroid cells but the precise binding site or whether it is recruited indirectly by other factors such as GATA-1 is not known (26,27). USF is a dimeric protein composed of either USF1 or USF2 and both homo- and heterodimers exist and bind to E-box elements (28). USF interacts with a conserved E-box element located 60 bp downstream of the adult β-globin transcription start site *in vitro* and can also be cross-linked to the promoter in erythroid cells (29,30). Mutations of the +60 E-box reduced transcription of the human β-globin gene *in vitro* (29). An additional conserved E-box is located immediately downstream of the transcription start site (29). This E-box overlaps with the initiator and also interacts with USF *in vitro*. Expression of a dominant negative mutant USF (AUSF), which dimerizes with USF proteins but does not bind DNA, reduces recruitment of RNA polymerase II (Pol II) at the β-globin gene locus (30). In addition, the expression of AUSF also resulted in impaired differentiation of erythroid cells in transgenic mice demonstrating a critical role of USF in adult β-globin gene regulation (31,32). 

*Cis*-element redundancy often presents a challenge in molecular biology especially when attempting to interrogate the functional potential of a *cis*-element *in vivo*. Removal of *cis*-regulatory elements by homologous recombination is feasible but laborious and changes the sequence context (33,34). Artificial DNA-binding proteins comprising zinc finger domains can surmount this challenge (35,36). Zinc finger DNA-binding domains (ZF-DBD) bind to DNA in a sequence-specific manner with high affinity and neutralize a target site (36). A single zinc finger domain can recognize three base pairs of DNA—an interaction that can be extended by adding additional zinc finger domains in tandem to allow the recognition of long asymmetric DNA sequences (37,38). The benefit of using ZF-DBDs includes rapid delivery and, more importantly, high resolution for *cis*-element detection. The high resolution allows targeting of sequences longer than 18 bp, long enough to bind ZF-DBDs with high affinity and specificity and to confer a unique signature within the genome (38). However, unspecific binding of ZF-DBDs to low-affinity DNA binding sites is a concern that may be addressed to some degree using ZF-DBDs without effector domains.

In this study, we designed and functionally characterized a ZF-DBD targeting and neutralizing the +60 E-box of the murine βmaj-globin gene. Expression of the +60 ZF-DBD led to a reduction in βmaj-globin gene expression in a murine erythroleukemia (MEL) cell line. We also observed a reduction in RNA Pol II recruitment at the βmaj-globin gene relative to vehicle control cells. The association of Pol II and transcription factor NF-E2, which is a tissue-restricted transcription factor previously implicated in Pol II recruitment at the β-globin gene locus (7), with LCR element HS2 was increased in cells expressing the +60 ZF-DBD. Furthermore, expression of the +60 ZF-DBD impaired conformational changes in the β-globin gene locus that establish proximity between the LCR and the adult βmaj-globin gene promoter. Expression of a negative control (NC) ZF-DBD targeting a putative neutral site located between the βmin- and the βmaj-globin genes had no effect on βmaj-globin gene transcription.

## MATERIALS AND METHODS

### Zinc finger design and construction

The +60 ZF-DBD and the NC ZF-DBD were designed and polymerase chain reaction (PCR) assembled as described previously (36,39). The ZF-DBDs harbored six zinc fingers that were designed using the ‘zinc finger tools’ Web site (Mandell and Barbas. Zinc finger (ZF) tools, version 3.0. www.scripps.edu/mb/barbas/zfdesignhome.php). The oligonucleotides used for assembly and amplification are listed in Supplementary Tables S1 and S2. Zinc finger domains 1–3 and 4–6 were assembled and amplified separately and ligated together with a linker region to construct the ZF-DBD coding fragment. We used the same assembly strategy PCR conditions as described by Barrow *et al.* (36). Sequences encoding for nuclear localization and FLAG tag were incorporated during the final amplification steps as outlined by Barrow *et al.* (36). The coding sequences for the ZF-DBDs were ligated into the HpaI site of the retrovirus vector pMSCV-neo (Clontech). The coding sequences for the +60 ZF-DBD and the NC ZF-DBD were sequenced before large-scale preparation of the plasmids.

### Cell culture and transfections/transductions

MEL and Phoenix A cells were grown in Dulbecco's modified Eagle's medium (Cellgro) containing 10% (vol/vol) fetal bovine serum (FBS) and 1% (vol/vol) penicillin/streptomycin (Cellgro). The cells were cultured at 37°C in the presence of 5% CO_2_ at a density of 2 × 10^5^ cells/ml. MEL cells were induced to differentiate in the presence of 2% (vol/vol) dimethyl sulfoxide (DMSO) for 72 h. For transfections, the pMSCV-neo vector or the pMSCV-neo vector containing the coding sequences for the +60 E-box or the NC ZF-DBD was transfected into the Phoenix A packaging cell line using Lipofectamine 2000 (Invitrogen) according to a protocol provided by the manufacturer. After 48 h incubation, cells were centrifuged and the supernatant containing replication-defective virus was treated with 2 μg/ml polybrene (Sigma) and added to MEL cells. After 48 h incubation, the transduced MEL cells were selected by incubation in the presence of 100 μg/ml geneticin (Cellgro). Single cell clones were selected by dilution of the pool of stably transduced MEL cells and seeding of single cells into 96-well culture dish. Colonies were then transferred to larger tissue culture dishes and expanded.

### Immunoblot analysis

Immunoblot analysis was performed as described in Barrow *et al.* (36). Nuclear and cytoplasmic proteins were isolated using the NE-PER kit (Thermo Scientific) according to the protocol provided by the manufacturer and published procedures (40). For immunoblot analysis, 10–20 μg of protein extracts from MEL cells or from cytoplasmic or nuclear fractions was loaded onto 4–15% (wt/vol) TGX Tris–HCl gels (Bio-Rad), separated by sodium dodecyl sulfate–polyacrylamide gel electrophoresis, transferred to polyvenylidenefluoride (PVDF) membranes and incubated with rabbit anti-ZF sera (a gift from Carlos Barbas, Scripps Research Institute, La Jolla, CA, USA), mouse anti-BRG1 (H-88, Santa Cruz, sc-10768), rabbit anti-USF2 (N-18, Santa Cruz, sc-861) or mouse anti-Tubulin (G-8, Santa Cruz, sc-55529). After several washes, the membranes were incubated with secondary anti-mouse or anti-rabbit antisera (Santa Cruz), and proteins were detected using enhanced chemiluminescence (ECL) reagent (Millipore) and visualized using X-ray films (Kodak).

### RNA isolation and quantitative reverse transcription-PCR analysis

RNA isolated from MEL cells using the RNeasy kit (Qiagen) was reverse transcribed using the iScript complementary DNA synthesis kit (Bio-Rad) and subjected to quantitative PCR analysis as described previously (36) using the following primers: mouse β-major, upstream (US) 5′-CACATTTGCTTCTGACATA-3′, downstream (DS) 5′-GCAGAGGCAGAGGATAGGTC-3′; mouse dematin, US 5′-ACCGCATGAGGCTTGAGAGG-3′, DS 5′-TCTTCTTAAGTTCGTTCCGCTTCC-3′; mouse β-spectrin, US 5′-GCTTAAGGAACGCCAGACTCCAG-3′, DS 5′-ATTTCTCCTGCTCGTCTTTGT-3′; mouse β-actin US 5′-GTGGGCCGCTCTAGGCACCA-3′, DS 5′-TGGCCTTAGGGTGCAGGGGG-3′; mouse GATA1 US 5′-CACTCCCCAGTCTTTCAGGTGTA-3′, DS 5′-GGTGAGCCCCCAGGAATT-3′; mouse Runx1 US 5′-CGGTAGAGGCAAGAGCTTCA-3′, DS 5′-GATGTCTTCGGGGTTCTCGG-3′; Mouse DOK2 US 5′-GAAGCTGCGATGGTCAGGAT-3′, DS 5′-GCCACTTCTTGCCAAAGGTC-3′; mouse Ldb1 US 5′-TGTGCCTGTCCTGGTTGTTC-3′, DS 5′-GTGGGTACATGGGAGTTGGG-3′; mouse ε/γ US 5′-GGCCTGTGGAGTAAGGTCAA-3′, DS 5′-GCAGAGGACAAGTTCCCAAA-3′; mouse glyceraldehyde 3-phosphate dehydrogenase (GAPDH) US 5′-CCAAGGTCATCCATGACAACT-3′, DS 5′-ATCACGCCACAGCTTTCC-3′. All reverse transcriptase-polymerase chain reaction data were normalized to mouse β-actin or mouse GAPDH levels. 

### Chromatin immunoprecipitation and ChIP-exonuclease sequencing

Chromatin immunoprecipitation (ChIP) assays were performed as described previously (36). Briefly, 2 × 10^7^ MEL cells were incubated in 2% formaldehyde for 10 min. After quenching the reaction with 0.125 M glycine for 5 min, cells were lysed and cross-linked chromatin was subjected to sonication using conditions yielding 200–500 bp fragments. The lysates were then incubated with mouse IgG (Santa Cruz; sc-2025) at 4°C for 2 h and precleared with protein A sepharose beads (GE healthcare; CL-4B). Precleared lysates were centrifuged at 1700*g* for 10 min at 4°C. The supernatant was incubated overnight at 4°C on a rotating wheel with the following specific antibodies: 10 μg of RNA-Pol II [C-terminal domain (CTD) 4H8; Millipore], RNA-Pol II PS2 (Abcam; Ab 5095), Tal 1 (C-21, Santa Cruz Biotechnology, sc-12984X), USF2 (N-18, Santa Cruz Biotechnology, sc-861X), Brg-1 (H-88, Santa Cruz Biotechnology, sc-10768X) or 10 μl of ZF antibody (a gift from Dr Carlos Barbas, Scripps Research Institute, California). After several washes with high and low salt buffers, the samples were subjected to phenol/chloroform/isoamylalcohol and chloroform extractions, and the DNA was precipitated by adding 2.5 × volume of 100% ethanol (vol/vol) and 10 μg glycogen (Invitrogen) overnight at −20°C. The DNA precipitates were washed with 70% ethanol (vol/vol), resuspended in 10 mM Tris-Cl, pH 8.5, and subjected to quantitative (q) PCR as described previously (36) using the following primers: mouse βmaj-globin, US 5′-AAGCCTGATTCCGTAGAGCCACAC-3′ DS 5′-CCCACAGGCCAGAGACAGCAGC-3′; mouse HS2 US 5′-TGCAGTACCACTGTCCAAGG-3′, DS 5′-ATCTGGCCACACACCCTAAG-3′; NC US 5′-CTAGAGACCCATGATTGA-3′, DS 5′-TCAATCATGGGTCTCTAG-3′; mouse βmaj 3′region, US 5′-GCTCTTGCCTGTGAACAATG-3′, DS 5′-TGCTTTTTATTTGTCAGAAGACAG-3′.

For ChIP-exonuclease sequencing (ChIP-Exo-Seq), we followed a protocol provided by Peconics and published by Rhee and Pugh (41). The MEL cell clone 15 expressing higher levels of the +60 ZF-DBD and the control cells harboring the vector only were subjected to cross-linking and sonication as described under the ChIP protocol. Sonication yielded fragments between 100 and 500 bp. For each cell clone 1.8 × 10^8^ million cells were processed and divided into nine aliquots representing ∼2 × 10^7^ million cells each. Three aliquots per antibody were sent to Peconics for ChIP-Exo-Seq. The immunoprecipitation step was performed using 5 μg of antibody per 10^7^ cells. The immunoprecipitated chromatin was then subjected to exonuclease treatment and high-throughput sequencing; these procedures were performed by Peconics. Bam-files and Bed-graph files provided by Peconics were analyzed using the IGV2.3 genome browser [http://broadinstitute.org, (42,43)].

### Chromatin conformation capture

The chromatin conformation capture (3C) assay was performed as described previously with minor modifications (8,44). Briefly, 4 × 10^7^ cells were cross-linked with 2% formaldehyde for 10 min and stopped by the addition of glycine at a final concentration of 0.125 M. Cells were pelleted, washed twice with cold phosphate-buffered saline and lysed in lysis buffer (10 mM Tris, pH8.0, 10 mM NaCl, 0.2% NP40 and protease inhibitors) at 4°C for 90 min while rotating. The nuclei were collected by centrifugation, washed with appropriate 1 × restriction enzyme buffer (Buffer 3, New England Biolabs. NEB) and then resuspended in restriction buffer 3 (NEB) containing 0.3% sodium dodecyl sulfate (SDS) at 37°C for 1 h while shaking. Triton-X-100 was added at a final concentration of 1.8% to sequester SDS, and incubation proceeded for another 1 h at 37°C. The chromatin was incubated with 300 U Bgl II (NEB) at 37°C overnight while shaking, and the reaction was stopped by adding SDS to a final concentration of 1.6% and incubating at 65°C for 30 min. The samples were subsequently diluted with ligation reaction buffer to a final volume of 1 ml. The ligation reaction buffer contained 1% Triton X-100, 50 mM Tris, pH 7.5, 10 mM MgCl_2_, 10 mM dithriotheitol (DTT), 0.1 mg/ml bovine serum albumin and 1 mM adenosine triphosphate. T4 DNA ligase (600 U, NEB) was added and the reaction was incubated at 16°C for 5 h followed by ligation for 30 min at room temperature. After reversal of the cross-link, the DNA was isolated, amplified and cloned into pCR4-TOPO vector (Invitrogen) for sequencing. To control for PCR amplification efficiency of different primer pairs, equimolar amounts of plasmids containing the PCR products were mixed together to generate the control templates. PCR reactions were performed with control and experimental templates amplified in parallel. To control for potential differences in template quality, the interactions at the β-globin locus from the experimental templates were normalized to interaction frequencies with the GAPDH locus, which were low and quantified with ImageQuant. The calculation of the relative cross-linking frequency between two loci was performed as described by Tolhuis *et al.* (8).

## RESULTS

**Figure 1.**
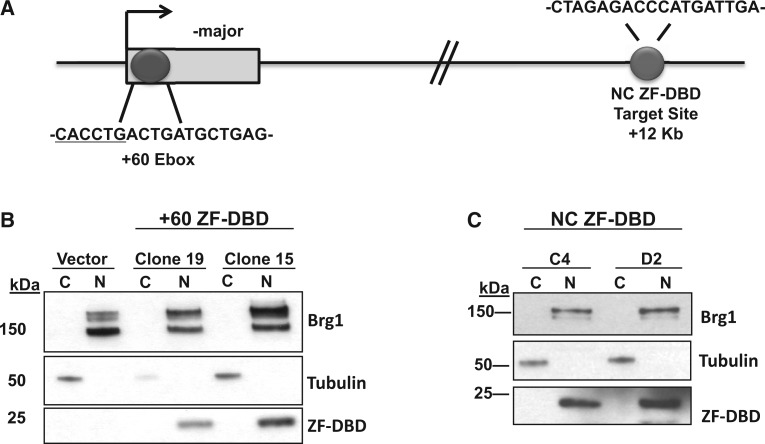
Synthetic ZF-DBDs localize to the nucleus in MEL cells. (**A**) Target sites for the NC and +60 ZF-DBDs are shown relative to the transcription start site of the βmaj-globin gene (not drawn to scale). (**B**) Compartmentalization immunoblot where cells were fractionated into cytosolic (C) and nuclear (N) fractions. Brg1 and tubulin (top and middle panel, respectively) represent controls. Two single cell clones reflecting low and medium protein expressing levels (clones 19 and 15, respectively) both localize the ZF-DBD to the nuclear fraction in MEL cells. (**C**) Compartmentalization immunoblot showing that the NC ZF-DBD protein in MEL cell populations C4 and D2 localized to the nucleus (bottom panel).

ZF-DBDs derived from the Barbas modules were designed to neutralize the +60 E-box or to bind a NC region located downstream of the βmaj-globin gene. The designed ZF-DBDs are composed of six zinc finger domains to target 18-bp regions harboring the target sites (Figure 1A). The coding region for the ZF-DBD together with FLAG and nuclear localization signal tags were ligated into the retroviral pMSCV vector. MEL cells were then transduced to generate stable populations for the NC ZF-DBD through geneticin sulfate selection. Single cell clones stably expressing either lower levels or higher levels of the +60 E-box ZF-DBD protein were selected to evaluate the effect of different concentrations of the ZF-DBD on transcription factor binding and βmaj-globin gene expression. The NC ZF-DBD was designed to interact with a putative neutral DNA sequence located in between the βmaj- and βmin-globin genes. The NC ZF-DBD served as a NC and was not expected to change expression of the adult βmaj-globin gene. The +60 ZF-DBD was designed to interact with an E-box located 60 bp downstream of the βmaj-globin transcription start site. We expected the +60 ZF-DBD to prevent the binding of transcription factor USF to this site and to reduce βmaj-globin gene transcription.

To confirm nuclear localization, MEL cells harboring the NC- and the +60 ZF-DBDs were fractionated into cytoplasmic and nuclear fractions (Figure 1B and C). The nuclear protein BRG1 and the cytoplasmic protein β-tubulin were used as controls to indicate complete fractionation. The data demonstrate that the ZF-DBDs localized to the nucleus and that there was no expression of ZF-DBDs in cells harboring the empty vector. Two single cell clones expressing the +60 ZF-DBD at different levels were isolated and expanded. The data show that clone 19 expressed lower levels of the +60 ZF-DBD compared with clone 15 (Figure 1B).

**Figure 2.**
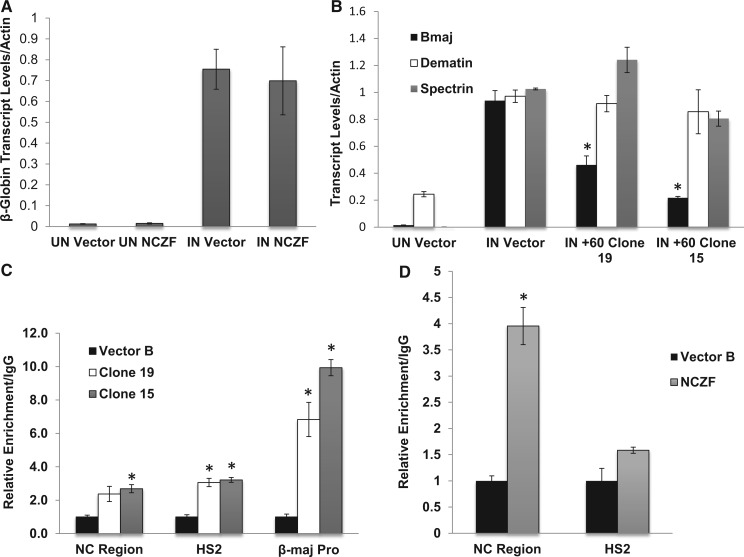
Expression of the +60 ZF-DBD reduces βmaj-globin gene expression. (**A**) The qPCR analysis of βmaj-globin gene transcription in uninduced (UN) or DMSO-induced (IN) MEL cells expressing the NC ZF-DBD (NCZF) or harboring the vector control (Vector). ****(**B**) The qPCR analysis of βmaj-globin, dematin and spectrin gene transcription in uninduced (UN) or DMSO-induced (IN) MEL cells harboring the vector (Vector) or expressing the +60 ZF-DBD (+60) in the two different clonal cell lines (clone 15 and 19). The data presented in (A) and (B) resulted from two independent RNA extractions with the qPCR performed in triplicate ±SEM. Statistical analysis was based on the Student’s *t*-test (*P < 0.05). (**C**) ChIP analysis of the occupancy of the +60 ZF-DBD to selected sites in the murine β-globin gene locus including the NC Region, LCR HS2 (HS2) and the βmaj-promoter (β-maj Pro). Two single cell clones expressing low (clone 19) or high (clone 15) levels of the +60 ZF-DBD or a pool of cells harboring the empty vector (Vector B) was analyzed. (**D**) ChIP analysis of NC ZF-DBD occupancy at the indicated regions in the β-globin locus. Vector B denotes control cells harboring the empty vector. The data in (C) and (D) represent two independent ChIP experiments with the PCR performed in triplicate ±SEM. Statistical analysis was based on Student’s *t*-test (*P < 0.05).

To determine the effect of the ZF-DBDs on adult βmaj-globin expression, RNA was extracted from uninduced and DMSO-induced MEL cells and reverse transcribed. Quantitative PCR analysis in stable MEL cells expressing the NC ZF-DBD revealed that this ZF-DBD did not affect βmaj-globin gene expression in either uninduced or DMSO-induced MEL cells (Figure 2A). In contrast, βmaj-globin gene expression significantly decreased in cells expressing the +60 ZF-DBD compared with vehicle control cells (Figure 2B). Furthermore, the decrease in βmaj-globin gene expression occurred in a dose-dependent manner relative to the concentration of the +60 ZF-DBD present (Figure 2B); clone 15, which expressed higher levels of the +60 ZF-DBD (Figure 1B) revealed lower levels of βmaj-globin gene expression compared with clone 19, which expressed lower levels of the protein. To analyze the specificity of the +60 ZF-DBD, additional E-box containing erythroid-specific genes such as dematin and β-spectrin were evaluated. The data show that expression of the +60 ZF-DBD did not affect expression of the control genes (Figure 2B). The data demonstrate that the +60 ZF-DBD specifically reduced expression of the adult βmaj-globin gene without affecting expression of other erythroid-specific genes.

Next to confirm the specificity of the ZF-DBDs, we examined target occupancy by ChIP in induced MEL cells. The +60 ZF-DBD associated with the βmaj-globin promoter, with a higher level of occupancy in clone 15 compared with clone 19 (Figure 2C). The association of the +60 ZF-DBD with the NC-region or LCR HS2 was lower. The NC ZF-DBD associated with the NC-region in the pool of transduced MEL cells (Figure 2D). We detected a low level of occupancy at LCR HS2 that was not statistically significant.

**Figure 3.**
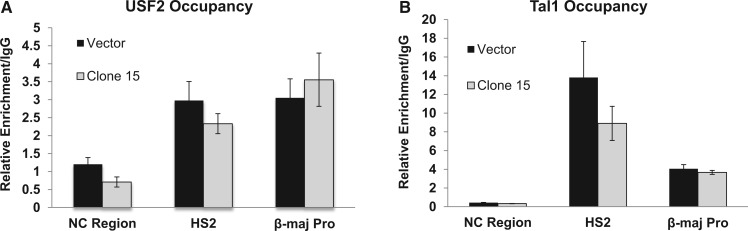
USF and Tal1 continue to associate with the βmaj-globin gene promoter in MEL cells expressing the +60 ZF-DBD. ChIP analysis of the occupancy of USF2 (**A**) or Tal1 (**B**) to select sites in the murine β-globin gene locus as indicated and described in Figure 2. MEL cell clone 15 expressing higher levels of the +60 ZF-DBD or a pool of cells harboring the empty vector (Vector) was analyzed. The data in (A) and (B) represent two independent ChIP experiments with the PCR performed in triplicate ± SEM. Statistical analysis was based on Student’s *t*-test.

To analyze the consequence of neutralizing the +60 E-box on the binding of transcription factors involved in βmaj-globin gene expression, we performed a series of ChIP experiments. First, we examined candidate transcription factors that are known to bind to E-box sequences such as USF1 and USF2 as well as Tal1. 8Both USF (Figure 3A) and Tal1 (Figure 3B) occupied both HS2 of the LCR and the β-major promoter in induced vehicle-treated control MEL cells. Surprisingly, the associations of USF2 (Figure 3A) and that of Tal1 (Figure 3B) with the adult βmaj-globin gene promoter were not affected in cells expressing the +60 ZF-DBD compared with vehicle control cells.

**Figure 4.**
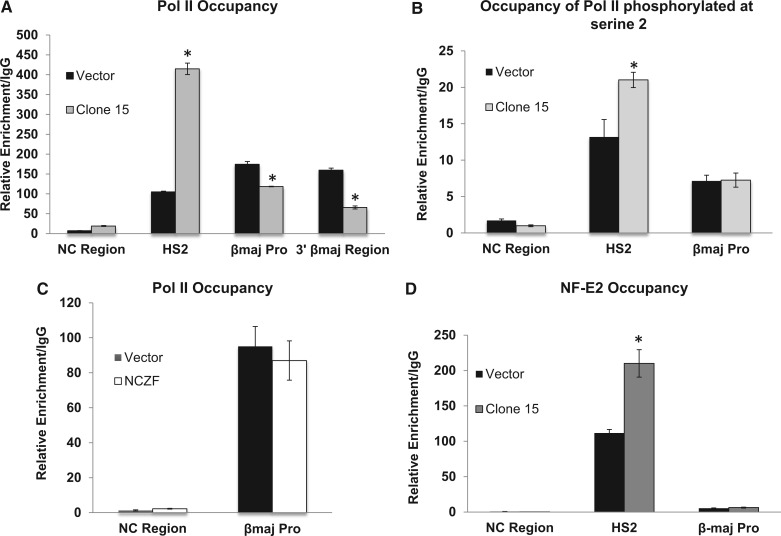
Decreased Pol II association at the βmaj-globin promoter and increased binding of Pol II and NF-E2 at LCR HS2 in MEL cells expressing the +60 ZF-DBD. (**A** and **B**) ChIP analysis of Pol II (A), Pol II phosphorylated at serine 2 (B) or NF-E2 (**D**) occupancy at select sites in the murine β-globin gene locus as indicated and described in Figure 2. The 3′βmaj-region reflects a DNA sequence located at the 3′end of the βmaj-globin gene. MEL cell clone 15 expressing higher levels of the +60 ZF-DBD or a pool of cells harboring the empty vector (Vector) was analyzed. (**C**) Occupancy of Pol II at the NC region and at the βmaj-promoter in vector control cells (Vector) or cells expressing the NC ZF-DBD as indicated. The data in A, B, C and D represent two independent ChIP experiments with the PCR performed in triplicate ±SEM. Statistical analysis was based on Student’s *t*-test (*P < 0.05).

The binding of the +60 ZF-DBD downstream of the transcription start site could interfere with the recruitment and/or the elongation of transcription complexes at the adult βmaj-globin gene promoter. To distinguish between these possibilities, we examined Pol II occupancy at the β-globin gene locus in induced MEL cells harboring the vector or the construct expressing the +60 ZF-DBD (Figure 4). We observed a decrease in the recruitment of Pol II at the adult βmaj-globin gene promoter in cells expressing the +60 ZF-DBD (Figure 4A). The decrease in Pol II occupancy was more pronounced at the 3′end of the βmaj-globin gene suggesting that expression of the +60 ZF-DBD affects both recruitment and elongation of Pol II. Interestingly, compared with control cells, cells expressing the +60 ZF-DBD revealed enhanced association of Pol II at LCR HS2. We next analyzed recruitment of Pol II phosphorylated at serine 2 of the CTD (Figure 4B). CTD serine 2 phosphorylation occurs during transcription elongation. Associations of the phosphorylated form of Pol II with LCR HS2 also increased in cells expressing the +60 ZF-DBD. We did not observe a decrease in association of the serine 2 phosphorylated form of Pol II with the βmaj-globin promoter showing that the defect in elongation is not due to a failure to phosphorylate the serine 2 residue of the Pol II C-terminal domain. The data strongly suggest that the binding of the +60 ZF-DBD at the βmaj-globin downstream promoter element interfered with the recruitment and elongation of transcription complexes at the β-globin gene promoter. Expression of the NC ZF-DBD did not affect recruitment of Pol II at the βmaj-globin gene promoter (Figure 4C), demonstrating that ZF-DBDs only exert effects if targeted to specific sites.

Previous studies have shown that transcription factor NF-E2 associates with LCR HS sites as well as with the βmaj-globin promoter and mediates the transfer of Pol II from the LCR to the β-globin gene (15,45,46). We examined the association of NF-E2 with LCR HS2 and with the adult βmaj-globin gene promoter in cells expressing the +60 ZF-DBD using ChIP (Figure 4D). The results show that expression of the +60 ZF-DBD increased the interaction of NF-E2 with LCR HS2, similar to what we observed for Pol II. The binding of NF-E2 at the βmaj-globin promoter was low, and changes in cells expressing the +60 ZF-DBD were not statistically significant.

**Figure 5.**
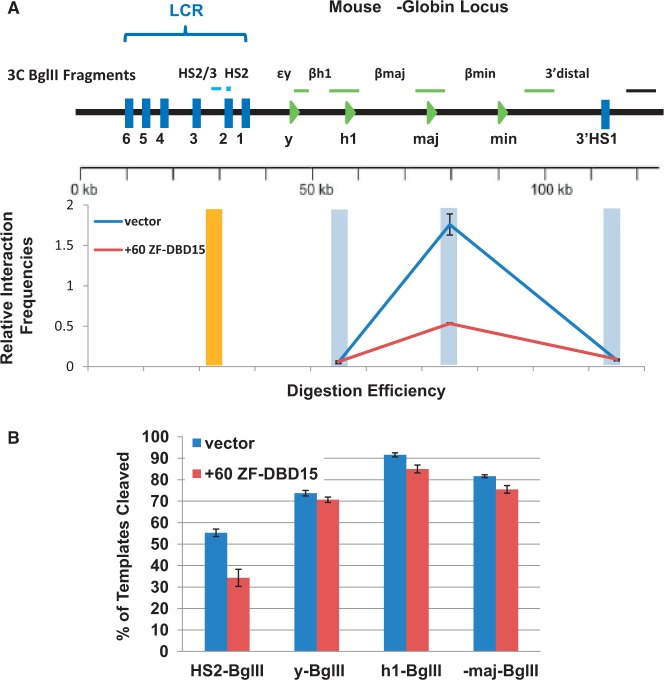
Expression of the +60 ZF-DBD impairs establishment of proximity between LCR HS2 and the βmaj-globin gene promoter. (**A**) Depiction of the murine β-globin gene locus. Black bars represent Bgl II restriction fragments analyzed by 3C in this study. The arrow indicates the relative locations of LCR HS2 and the βmaj-globin gene promoter. The graph depicts the results of 3C analysis of interaction frequencies between LCR HS2 and three regions in the murine β-globin gene locus as indicated. The single cell clone 15 expressing higher levels of the +60 ZF-DBD or a pool of cells harboring the empty vector (Vector) was analyzed. The data represent two independent 3C experiments with the qPCR performed in triplicate ±SEM. (**B**) Relative digestion efficiency at Bgl II restriction sites in the β-globin gene locus as indicated. Digestion efficiency was determined by quantitative PCR with an uncross-linked BAC containing the murine β-globin gene locus used as a positive control (efficiencies of digestion in the bacterial artificial chromosome (BAC) were set at 100%).

Next, we examined proximity between the adult βmaj-globin promoter and the LCR using the 3C procedure (Figure 5A). It has been shown previously that the β-globin locus undergoes a conformation change that juxtaposes the βmaj-globin promoter and the LCR in cells in which the gene is expressed (8,9). We performed the 3C assay in cells expressing the vehicle only and in the clonal +60 ZF-DBD cell line 15. The data in Figure 5B show that Bgl II digestion efficiency at the various regions of the β-globin locus analyzed in the 3C experiment was similar in the two different cell lines analyzed. The 3C data shown in Figure 5A reveal a reduced interaction between LCR HS2 and the adult βmaj-promoter in induced MEL cells expressing the +60 ZF-DBD. Essentially no interactions were observed between the LCR and the βH1 gene, which is not expressed in MEL cells, or between the LCR and a region far downstream of the βmaj-globin gene.

**Figure 6.**
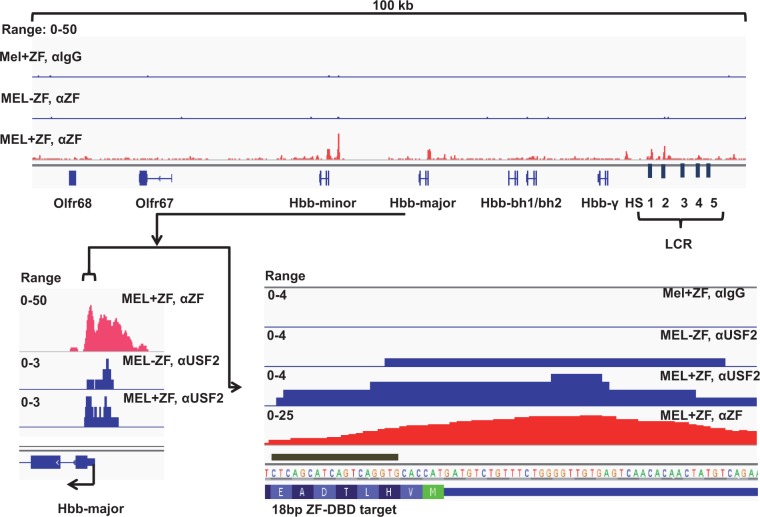
ChIP-Exo-Seq analysis of +60 ZF-DBD and USF2 occupancy in the β-globin gene locus in MEL cells. MEL cells expressing the +60 ZF-DBD (MEL+ZF) or MEL cells not expressing a ZF-DBD (MEL−ZF) were subjected to ChIP-Exo-Seq using antibodies against the ZF-DBD background (αZF), USF2 (αUSF2) or NC IgG (αIgG). Bed-graph files were analyzed using the Integrated Genomics Viewer (42,43). Shown on top is the mouse β-globin gene locus and flanking olfactory receptor (Olfr) genes. On the lower left is a magnified view of the adult βmaj-globin gene showing ChIP-Exo-Seq patterns for the +60 ZF-DBD and USF2. Further magnification showing the DNA sequence of the adult βmaj-globin downstream promoter region is shown on the lower right. The black bar indicates the target sequence of the +60 ZF-DBD.

To address the specificity of the +60 ZF-DBD and to examine if binding of the +60 ZF-DBD alters the interaction pattern of USF with the βmaj-globin gene promoter, we subjected MEL cells expressing or not expressing the ZF-DBD to ChIP-Exo-Seq (Figure 6). ChIP-Exo-Seq is related to ChIP-Seq but contains an additional step in which accessible DNA is digested with exonuclease leaving chromatin fragments that are bound by proteins and thus protected from exonuclease digestion (41). We used three different antibodies in these experiments, a NC IgG antibody, an antibody specific for the ZF-DBD backbone and an antibody specific for USF2 (Figure 6). The data demonstrate that the ZF-DBD antibody is highly specific; it did not yield any peaks above the IgG background in cells not expressing the +60 ZF-DBD. Importantly, cells expressing the +60 ZF-DBD revealed two prominent peaks associated with the adult βmaj- and βmin-globin gene promoters. The βmin- and βmaj-globin downstream promoters contain identical +60 ZF-DBD target sequences. Strikingly, the ChIP-Exo-Seq pattern for the +60-ZF-DBD in the βmaj-globin downstream promoter region demonstrates that protection from exonuclease digestion starts immediately at the 3′ end of the targeted sequence as indicated by the black bar in the lower right of Figure 6. This demonstrates that the +60-ZF-DBD was interacting specifically with the targeted sequence. The increased sequence reads over a broad range of the promoter, and upstream regions likely reflect binding of other proteins that are cross-linked together with the +60 ZF-DBD at the adult βmaj-globin promoter. We also subjected MEL cells expressing or not expressing the +60 ZF-DBD to ChIP-Exo-Seq using antibodies against USF2. Expression of the +60 ZF-DBD changed the USF2 ChIP-Exo-Seq pattern at the adult βmaj-globin gene, suggesting an altered interaction mode of USF2 containing protein complexes with the adult globin gene promoter (Figure 6).

**Figure 7.**
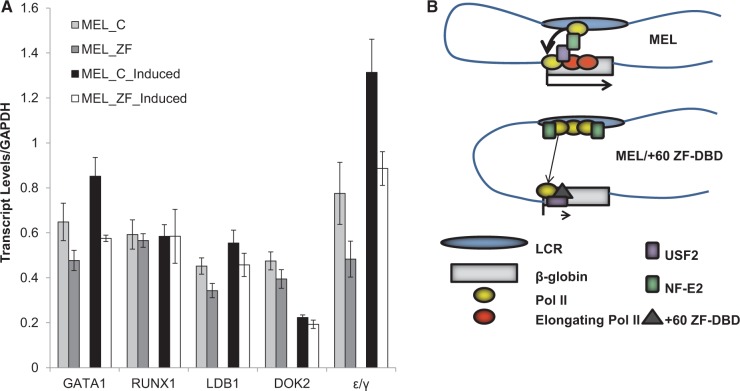
Expression analysis of erythroid genes in MEL cells expressing or not expressing the +60 ZF-DBD and model of the impact of the ZF-DBD on transcription complex assembly and function at the adult βmaj-globin gene. (**A**) The qPCR analysis of ε/γ-globin, GATA1, Runx1, Ldb1 and DOK2 gene transcription in uninduced (MEL) or induced (MEL-induced) MEL cells expressing (MEL_ZF) or not expressing (MEL_C) the +60-ZFDBD. The data resulted from two independent RNA extractions with the qPCR performed in triplicate ±SEM. Statistical analysis was based on the Student’s *t*-test. None of the changes in messenger RNA levels were statistically significant (*P* > 0.05). (**B**) Model for the impact of the +60 ZF-DBD on transcription complex assembly and function at the adult βmaj-globin gene.

There were additional +60 ZF-DBD peaks associated with the β-globin gene locus, one prominent peak upstream of the β-minor globin gene and three peaks upstream of the embryonic ε/γ-globin gene, two of which corresponding to LCR elements HS1 and HS2 (Figure 6, top). Genome wide the +60 ZF-DBD interacted with other sites mostly located in intergenic regions or in introns. Most of these interactions are likely due to the presence of low-affinity binding sites or due to opportunistic residence at highly accessible regions at which lack of competition led to relatively high cross-linking frequencies. We examined expression of five additional genes that were associated with +60 ZF-DBD binding peaks in intronic or upstream promoter regions, including the embryonic ε/γ-globin, Runx1, Ldb1, DOK2 and GATA1 genes (Figure 7A). None of these genes revealed changes in expression levels between ZF-DBD expressing and non-expressing cells that were comparable with the reduction in expression of the adult βmaj-globin gene (Figures 2B and 7A).

## DISCUSSION

In this study, we examined the functional role of the adult βmaj-globin downstream promoter region using an artificial ZF-DBD targeting an E-box. Using artificial ZF-DBDs has several advantages over recombination-based strategies including high resolution and the ability to titrate the ZF-DBD so that either partial or complete elimination of the function of a *cis*-regulatory DNA element can be achieved. However, one concern of applying artificial ZF-DBDs for modulating gene expression is the fact that they can bind to unspecific DNA sequences harboring partial sequence homology to the targeted sequence. Therefore, we use ZF-DBDs without effector domains to minimize unspecific effects (36). We demonstrate in this study that a ZF-DBD targeting the +60 E-box interferes with the recruitment of Pol II to the βmaj-globin gene promoter and reduces transcription of that gene. The data show that the negative effects exerted by the +60 ZF-DBD were sensitive to the expression levels of ZF-DBD expression. A ZF-DBD targeting a NC region far downstream of the βmaj-globin gene had no effect on gene expression demonstrating that ZF-DBDs have to be targeted to specific sites to exert a function.

The ZF-DBDs used in this study harbored six ZFs specifying 18-bp DNA target sequences. Much like transcription factors in general, artificial ZF-DBDs interact with other sites with lower affinity. To determine the extent of ZF-DBD interactions in the MEL cells, we subjected +60 ZF-DBD expressing cells to ChIP-Exo-Seq (Figure 6). The data show specific interactions of the ZF-DBD with the adult β-globin gene promoters. However, the +60 ZF-DBD interacts with other sites in the MEL genome. Most of these sites are located in introns or intergenic regions and do not always reveal sequence similarity to the targeted sequence. We believe that most of the chromatin interactions of the ZF-DBD in MEL cells are due to the presence of low-affinity binding sites or caused by prolonged occupancy of highly accessible regions in pursuit of finding a binding site. Lack of competition would lead to efficient cross-linking of the ZF-DBDs at these sites. We examined several genes that revealed binding peaks for the +60 ZF-DBD in promoter or intronic regions, including genes encoding GATA1, Runx1, Ldb1 and Dok2 (Figure 7A). The +60 ZF-DBD did not change expression of any of these genes in MEL cells demonstrating that the binding of the ZF-DBD at most sites is without functional consequence. Nevertheless, the data highlight the importance of using ZF-DBDs without effector domains.

Previous studies have identified several DNA sequence elements in the adult β-globin downstream promoter region that contribute to efficient transcription. A sequence around +20 has been shown to mediate interactions with the TFIID complex, whereas the +60 E-box interacts with USF1 and two heterodimers (29,47). Both of these elements contribute to efficient *in vitro* transcription of the human β-globin gene. There is another E-box located immediately downstream of the transcription start site that interacts with USF1 *in vitro* (29). However, this site was not important for *in vitro* transcription of the human β-globin gene (29). Importantly, USF has also been shown to associate with the murine βmaj-globin gene promoter in the context of erythroid cells (30,44). Furthermore, expression of a dominant negative form of USF decreased transcription complex recruitment and βmaj-globin gene expression in murine MEL cells and in transgenic mice (30,31). The collective data suggest that USF interacts with the +60 E-box and mediates the recruitment of transcription complexes to the adult β-globin gene promoter. In support of this conclusion, expression of the +60 ZF-DBD in MEL cells reduced recruitment of Pol II and transcription of the βmaj-globin gene. However, we did not observe a decrease in the binding of USF at the adult βmaj-globin gene promoter in cells expressing the +60 ZF-DBD. One possible explanation for this observation is that Tal1, a hematopoietic-specific E-box binding protein, mediates the positive role of the +60 E-box in erythroid cell. However, the association of Tal1 with the adult βmaj-globin gene promoter was also not impaired in cells expressing the +60 ZF-DBD (Figure 4B). To examine in more detail if binding of the +60 ZF-DBD altered the interaction pattern of USF2 with the adult βmaj-globin gene promoter, we subjected the cells to ChIP-Exo-Seq (Figure 6). The ChIP-Exo-Seq pattern of USF2 at the adult β-globin promoter was different in cells expressing the +60 ZF-DBD compared with control MEL cells, suggesting an altered binding conformation of USF2 containing protein complexes. It is possible that the reduced binding and activity of Pol II at the adult βmaj-globin gene promoter in cells expressing the +60 ZF-DBD allows USF2 and associated proteins to bind to the E-box at the transcription start site (TSS) (29).

Regardless of how the +60 ZF-DBD interferes with the activity of USF, its binding at the βmaj-downstream promoter reduced efficient recruitment and elongation of transcription complexes. We and others previously hypothesized that transcription complexes are first recruited to the LCR, which is an extremely accessible region and contains a high density of transcription factor binding sites (48,49). Pol II can be cross-linked to LCR HS elements during erythroid differentiation before it is detectable at the β-type globin gene promoters (12,13). Pol II transcription complexes may thus be assembled at the LCR and then transferred to the globin gene promoter. USF and the erythroid transcription factor NF-E2 have been implicated in the transfer of Pol II from the LCR to the adult β-globin gene promoter (12,15,49). The data presented in this study demonstrate that although Pol II recruitment to the βmaj-globin gene promoter is impaired, the binding of Pol II at LCR HS2 is increased in cells expressing the +60 ZF-DBD. This supports the hypothesis that the LCR is the primary site of transcription complex recruitment. Interestingly, we also detected an increase in the association of NF-E2 with LCR HS2 in cells expressing the +60 ZF-DBD. The binding of NF-E2 at the promoter was low, and possible changes in binding at this site were not statistically significant. Nevertheless, the data provide further support of a model in which Pol II is transferred from the LCR to the adult β-globin gene promoter in a process facilitated by USF and NF-E2 (15,49). We had previously shown that USF and NF-E2 physically interact with each other in erythroid cells (15). In a recent study, examining chromatin accessibility using a high resolution DNase-I *in vivo* footprinting assay, investigators provided evidence suggesting that NF-E2 and USF interact with each other and that NF-E2 recruits USF indirectly to distal elements, whereas USF recruits NF-E2 indirectly to sites in the vicinity of transcription start sites (50). ChIP-Exo-Seq yielded a low number of sequence reads for USF2 selected βmaj-globin fragments. Perhaps, USF only interacts transiently with the βmaj-downstream promoter to recruit the transcription complex from the LCR and to stimulate early transcription elongation. NF-E2 could reach out from the LCR to interact with USF2 and this could facilitate the transfer of Pol II. The binding of the +60 ZFDBD could specifically interfere with the precise architecture of USF2/NF-E2-mediated interactions and consequently recruitment of transcription complexes (Figure 7B). 

Many recent studies have demonstrated that the β-globin gene locus changes conformations during differentiation of erythroid cells and activation of the globin genes (8,9,21,24). How proximity between the LCR and the adult β-globin gene is established is not known but requires proteins known to interact with GATA-1 or the GATA-1/Tal1 protein complex, Fog1 and LdB1 (21,24). In addition, the histone arginine methyltransferase Prmt1, which is recruited to the adult βmaj-globin gene promoter by USF, has been shown to be required for establishing proximity between the LCR and the adult globin promoter (44). Finally, the adenosine triphosphate-dependent chromatin remodeling enzyme Brg1 has also been shown to mediate proximity between the adult β-globin gene and the LCR (51). We demonstrate that the binding of the +60 ZF-DBD to the downstream promoter interfered with the recruitment of Pol II and establishment of proximity between the LCR and the βmaj-globin gene promoter. All of the proteins involved in mediating proximity between the LCR and the adult β-globin gene promoter also reduce the recruitment of transcription complexes to the gene (21,24,44,51). Perhaps what mediates interactions between the distal element and the promoter is the transfer of transcription complexes, an event that may be captured by the cross-linking reaction. This would be consistent with recent data suggesting that the fraction of erythroid cells exhibiting interactions between the LCR and the adult β-globin gene promoter in 3 C assays is low (52).

Grosveld *et al.* recently demonstrated that Pol II transcription initiates within transcription factories and that the elongating polymerase moves away from these loci (53). Furthermore, Groudine *et al.* showed that the LCR is required to anchor the β-globin gene locus at transcription factories and also mediates efficient transcription elongation of the β-globin gene (54). Our data suggest that the binding of the +60 ZF-DBD interferes with the LCR-mediated transfer of Pol II to the β-globin promoter and with the release of Pol II from the promoter. These events, however, are linked because if there is a deficiency in elongation, fewer Pol II transcription complexes are recruited to the promoter and accumulate at the LCR. Thus, our data strongly support the hypothesis that β-globin promoter associated Pol II is recruited from the LCR (48,49).

In summary, the current study demonstrates that targeting a ZF-DBD to the adult β-globin downstream promoter interferes with the recruitment and activity of Pol II. Thus, ZF-DBDs are useful tools for changing gene expression patterns.

## SUPPLEMENTARY DATA

Supplementary Data are available at NAR Online.

## FUNDING

National Institutes of Health (NIH) [RO1 DK083389 and R01 DK 052356 to J.B.; RO1 HL 090589 and RO1 HL 091929 to S.H.]. UF alumni fellowship and NIH supplement [R01 DK 052356 16-S1 to J.B.]. Funding for open access charge: National Institutes of Health [RO1DK083389] and overhead account.

*Conflict of interest statement*. None declared.

## Supplementary Material

Supplementary Data
