# Supplementary material for: Dissecting the function of the adult β-globin downstream promoter region using an artificial zinc finger DNA-binding domain
Source: Nucleic Acids Res. 2014 Jan 31;42(7):4363–74. doi: 10.1093/nar/gku107 (PMC3985677; doi:10.1093/nar/gku107)
Supplement: Supplementary Data [file supp_42_7_4363__index.html]

Dissecting the function of the adult β-globin downstream promoter region using an artificial zinc finger DNA-binding domain — Dissecting the function of the adult β-globin downstream promoter region using an artificial zinc finger DNA-binding domain — Supplementary Data 

# Dissecting the function of the adult β-globin downstream promoter region using an artificial zinc finger DNA-binding domain

## Supplementary Data

files

**Files in this Data Supplement:**

- Supplementary Data - docx file
