# Supplementary material for: Dissecting the function of the adult β-globin downstream promoter region using an artificial zinc finger DNA-binding domain
Source: Nucleic Acids Res. 2014 Jan 31;42(7):4363–74. doi: 10.1093/nar/gku107 (PMC3985677; doi:10.1093/nar/gku107)
Supplement: Supplementary Data [file supp_gku107_nar-03590-x-2013-File009.docx]

| Primer Nomenclature | Constant Oligos (5’-3’) | |
| --- | --- | --- |
| C1 (ZF 1-3) | CGGGGAGAAACCCTATAAGTGTCCGGAGTGTGGCAAGTCGTTCTC | |
| C1 (ZF 4-6) | TATAAGTGTCCGGAGTGTGGCAAGTCGTTCTC | |
| C2 | GCGTACCCACACGGGCGAAAAGCCGTACAAATGCCCAGAATGCGGTAAATCCTTCAGC | |
| C3 | CGGGGAGAAACCCTATAAGTGTCCGGAGTGTGGCAAGTCGTTCTC | |
|  | | General Primers (5’-3’) |
| Forward Primer (ZF1-3) | CAGGACGAATTCACTCGAGCCCGGGGAGAAACCCTATAAG | |
| Forward Primer (ZF4-6) | CAGGACGGATCCTATAAGTGTCCGGAGTGTGGCAA | |
| Reverse Primer (ZF1-3) | CAGGACAAGCTTGTGAGTGCGCTGGTG | |
| Reverse Primer (ZF4-6) | CAGGACGGTACCGCTGGTTTTTTTGCCGGTGTGAGTGCGCTGGTG | |
| Eukaryotic Forward Primer | ACGGTTAACATGCCGAAAAAAAAACGCAAAGTGCTCGAGCCCGGGGAGAAAC | |
| Eukaryotic Reverse Primer | GCAGTTAACTCACTTGTCATCGTCGTCCT | |

Table S1: Constant Primers and Oligonucleotides Used in Zinc Finger Construction

Oligonucleotides and primers used for the construction of the ZF-DBDs. Sequences are listed in the 5’-3’ orientation.

| Primer Nomenclature | Variable Oligos (5’-3’) |
| --- | --- |
|  | Oligonucleotides for +60 ZF-DBD |
| V1 (GAG) (ZF1-3): | GCCCGTGTGGGTACGCTGATGGCGCACCAGGTTATCGCTGCGCGAGAACGACTTGCCACAC |
| V2 (GCT) (ZF1-3): | CCTGTATGCGTCCGTTGATGGCGCACCAGTTCGCCGCTGGTGCTGAAGGATTTACCGCAT |
| V3 (GAT)(ZF1-3): | TTGTGAGTGCGCTGGTGGCGCACCAGGTTGCCGCTGGTACTAAAACTCTTCCCACATTCG |
| V1 (ACT) (ZF4-6): | GCCCGTGTGGGTACGCTGATGGCGAATCAGATCCAGATGGGTCGAGAACGACTTGCCACAC |
| V2 (CTG) (ZF4-6): | CCTGTATGCGTCCGTTGATGTTCGGTCAGCGCATCGTTGCGGCTGAAGGATTTACCGCAT |
| V3 (CAC)(ZF4-6) | CGGTGTGAGTGCGCTGGTGTTCGGTCAGCGCTTTTTTGCTACTAAAACTCTTCCCACATTCG |
|  | Oligonucleotides for NC ZF-DBD |
| V1 (GAG) (ZF1-3): | GCCCGTGTGGGTACGCTGATGGCTCGCCAGATGGCCCGCCTGCGAGAACGACTTGCCACAC |
| V2 (GCT) (ZF1-3): | CCTGTATGCGTCCGTTGATGGCGCACCAGGTTGCCGCTGGTGCTGAAGGATTTACCGCAT |
| V3 (GAT)(ZF1-3): | TTGTGAGTGCGCTGGTGTTCGGTCAGGTTGCCGCTGGTACTAAAACTCTTCCCACATTCG |
| V1 (ACT) (ZF4-6): | GCCCGTGTGGGTACGCTGATGGCGGGTCAGATCTTTTTTATCCGAGAACGACTTGCCACAC |
| V2 (CTG) (ZF4-6): | CCTGTATGCGTCCGTTGATGGCGCACCAGGTTATCGCTGCGGCTGAAGGATTTACCGCAT |
| V3 (CAC)(ZF4-6): | CGGTGTGAGTGCGCTGGTGTTCGGTCAGGGTGCTGTTCTGACTAAAACTCTTCCCACATTCG |

Table S2. Variable Oligonucleotides Used in Zinc Finger Construction

Oligonucleotides encoding the variable regions for ZF1-3 and ZF 4-6 of the ZF-DBDs targeted towards the +60 and +12 KB (NC) *cis*-elements are shown. All sequences are displayed in the 5’-3’ orientation.
